# Thermodynamic method for establishment of relationship between icephobicity/superhydrophobicity and microstructure–Based on computing for adhesion work

**DOI:** 10.1016/j.mex.2019.02.019

**Published:** 2019-02-28

**Authors:** H.Y. Zhang, H. Long, Y.L. Yang, J.F. Pan, L.S. Huang, X.K. Zhang

**Affiliations:** a School of Big Data Engineering, Kaili University, Kaili, 556011, China; b Key Laboratory and Innovative Teamwork of Low Dimensional Materials and Application Technology of Ministry of Education, School of Materials Science and Engineering, Xiangtan University, Xiangtan, 411105, China

**Keywords:** Computation of the adhesion work based on simulation for hierarchical structure of superhydrophobic surface, Thermodynamics, Superhydrophobicity, Adhesion work, Icing, Micro/Nanostructure

## Abstract

Superhydrophobic surfaces (SHS) have potential in solving the icing of aircraft, high-voltage overhead transmission lines, and other power network devices exposed to the air. For this reason, we wish to establish the relationship between microstructure and the adhesion work by thermodynamic method, also for analysis of the relationship between the hydrophobicity and icephobicity (or anti-icing). Therefore, respectively considering Cassie-Baxter and Wenzel states, such relationship was theoretically established based on one/two-step surface model, enlightened by natural and artificial SHS. Among it, how to obtain the adhesion work of icing per unit ice-solid interface is the key to this study. Followed by it, hydrothermal experiment, chemical deposition, and etching methods were performed to verify our theoretical results.

• How to model for the SHS based on the natural and artificial SHS;
• Computation for adhesion work (w_aw_) per unit area of a water droplet–SHS interface;
• Computation for adhesion work (w_ai_) per unit area of a frozen water droplet–SHS interface;
• Computation for reduced adhesion work (w_a2_) after icing;
• Hydrothermal experiment, chemical deposition and etching methods were used for validation of modeling.

## Graphical abstract

Flow chart of study for comparison between icephobicity and superhydrophobicity. The line of study shows such a process: from enlightenment, modeling, theoretical analysis, computing, visualization, to analysis for results.

**Figure:**
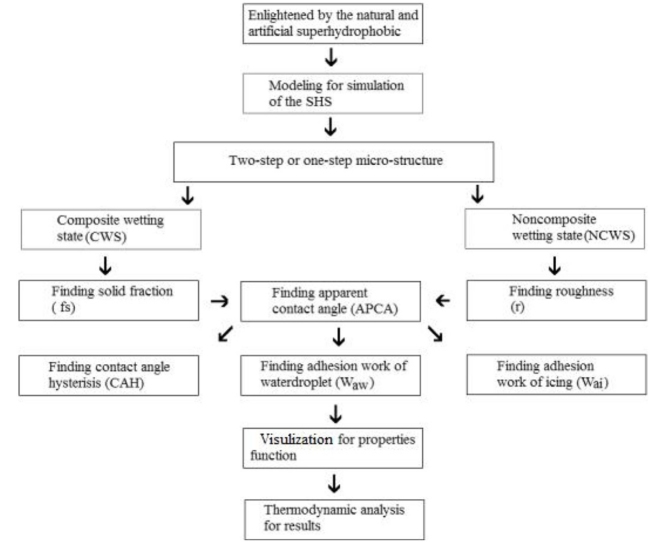

**Specifications Table**

**Table:** 

**Subject Area:**	*Materials Science* *Chemical Engineering*
**More specific subject area:**	*Materials surface and interface* *Composite and non-composite wetting state* *Adhesion work of interface*
**Method name:**	*Computation of the adhesion work based on simulation for hierarchical structure of superhydrophobic surface*
**Name and reference of original method:**	[2] D. Sarkar, M. Farzaneh, Superhydrophobic coatings with reduced ice adhesion, J. Adhes. Sci. Technol.23(9) (2009) 1215-1237. This study gave a good description for some superhydrophobic surfaces with specific topography to mitigate the ice accretion on power network equipment and other exposed structures by reducing ice-to-surface adhesion. The main factor owes to less solid-liquid contact area, thus decreasing heat transformation, effectively delaying the time to ice. The authors provided a experimental base for the superhydrophobic surfaces designed for anti-icing. According to their results of ice adhesion testing, if not coating a layer of low surface energy materials, merely creating a micro/nano binary structure is not enough, whether for the superhydrophobicity or for icephobicity. Otherwise a roughness surface (e.g., Etched Al, ZnO/Etched Al) even shows a stronger adhesion of ice than smooth surface (e.g., As-received Al) due to the mechanical intelocking. Therefore, it is necessary for the superhydrophobicity or for icephobicity to integrate micro/nano structure with low surface energy coatings (i.e., passivated by fluoro-alkyl silanes). Especially for the superhydrophobic surface designed for anti-icing, we must consider specific topography capble of keeping Cassie-Baxter and avoiding the mechanical intelocking. Consequently, we also should keep a reservation on the superhydrophobic surface for anti-icing. Inspired by the bove idea, we managed to study the above problem, namely theoretically explaining how some specific superhydrophobic surface can reduce the adhesion of ice to itself (here, not including the mechanical interlocking). Therefore, we proposed the thermodynamic method to discuss such a problem from the angle of the change in surface or interface free energy, namely the adhesion work (W_aw_ or W_ai_) from the solid-liquid or solid-solid interfaces. [3] A. Dotan, H. Dodiuk, C. Laforte, S. Kenig, The relationship between water wetting and ice adhesion, J. Adhes. Sci. Technol. 23(2009)1907-1915. Once more, the authors’ research provided immediately evident with the ultra-hydrophobic surface coated by polycarbonate to decrease ice adhesion by an 18 fold compared to the untreated aluminum surface(the best results), thereby they developed such an idea of solveing the icing of aircraft by using ice repellent surfaces (ice-phobic). Meanwhile, they studied the relationship between water wettability and ice adhesion by experiment, and come to a conclusion that the higher the contact angle, the lower is the adhesion of ice. For this reason, we also make a further study to theoretically establish the relation between the adhesion work (characterization for the icephobicity or anti-icing property) and surface microstructure based on the superhydrophobic surface, respectively considering the composite (Cassie-Baxter state) and non-composite wetting state (Wenzel state), and make a comparion between hydrophobicity and icephobicity (or anti-icing). [7] B. Li, Brief survey of deicing/anti- icing fluid and techniques for aircraf, *Cleaning World***28**(2012)26-31. To solve the icing on the aircraft, people proposed different methods for deicing/anti-icing. Author gave a brief survey of deicing/anti- icing fluid and techniques. Considering the cost and potentials, as well as the superhydrophobic surfaces, commonly regarded as an effective measure for anti-icing based on a series of anti-icing experiment, we also think now that such a surface has apparent advantage in anti-icing, we wish to make a study on how to tailor or design such a surface with appropriate topography, and roughness in detail, for the reference of surface micro-machining. [13] F. Wang, C. Li, Y. Lv, F. Lv, Y. Du, Ice accretion on superhydrophobic aluminum surfaces under low-temperature conditions, cold regsci technol. 62 (2010)29-33. To reduce ice formation on their aluminum surface for high voltage overhead transmission lines, authors coated its surface firstly with hydrophobic room temperature vulcanized silicone rubber (RTVSR), then with the low surface-energy stearic acid, thus obtained superhydrophobic surface with micronanoscale structure. Such a superhydrophobic surface has icephobic property, which did resist ice formation but was covered by a layer of ice after 30 min of spraying supercooled water Compared with bare hydrophilic aluminum surfaces. Their experiment results once again provided a evidence of the superhydrophobic surface for anti-icing, which can largely prevent ice formation on the surface except a few ice growth spots at a working temperature of − 6 °C. In a word, the present experiment indicates that the superhydrophobic surfaces surely have profound potential in solving the icing problem of aicraft, high-voltage overhead transmission, and the other power devices exposured to the air (low temperature and high humidity environment). Therefore, significant experiment enlights us a new line of how to theoretically establish a relation between the hydrophobicity and icephobicity for the superhydrophobic surface. [37] P. Guo, Y. Zheng, M. Wen, C. Song, Y. Lin, L. Jiang, Icephobic/anti-icing properties of micro/nanostructured surfaces, Adv. Mater.24(2012)2642-2648. Similarly, they did series of experiment to deeply study the Icephobic/anti-icing properties of hydrophobic surface. As is known, for a hydrophobic surface, their hydrophobicity mainly attributes to two aspects of micro/nanostructure and low surface energy coatings. Furthermore, for micro/nanostructure, it might be in micro scale, or nano scale, or their combination. Thereby authors made a comparison among them in Icephobic/anti-icing properties. The expleriment temporarily showed the advantage of the micro/nano combination structure, then nanostructure, lastly microstructure or smooth surface by comparing delaying time of the icing at a temperature of −10 °C. Based on their research, we continue to do this work from angle of theoretical study. Therefore, we respectively designed a one-step and two-step surface models to simulate superhydrophobic surface, and compared their adhesion work or anti-icing property, as our main study contents. [43] K. Chen, T. Sun. Effects of microstructure design on aluminum surface hydrophobic and ice-retarding properties, Asia-Pacific Journal of Chemical Engineering.12(2)(2017)307–312. This study further demonstrated that, for the superhydrophobic surfaces with micro/nanostructure, they showed difference in icephobicity or anti-icing property due to different microtopography. This also confirms that not all surface with microstructure or hydrophobicity have excellent icephobicity or anti-icing property. Only those surfaces with specific microtopography still capable of capturing air and keeping Cassie–Baxter wetting state after many times icing/de-icing cycles are still possess of outstanding icephobicity or anti-icing property. Authors designed a special microstructure with interconnected hollow honeycomb capable of meeting the above mentioned needs, and improving the mechanical strength. As a result, we also think that our study results are more likely applied to such a special micrstructure than a general microstructure. Certainly, for convenience of study, we designed a pillar model as a reference. [67] C. Laforte, J. Laforte. J. Carrier, How a solid coating can reduce the adhesion of ice on a structure, 6(2002)1-5. This paper aimed at the study for the the adhesion reduction efficiency of seven solid icephobic coatings. They found that the factor most affecting ice adhesion is roughness, and best explained the strong dependence of adhesion to roughness using mechanical adhesion mechanisms. Thus the most efficient of 37% to reduce adhesion was a compacted powder due to relatively weak mechanical interlocking. Referring to their experimental achievement, we managed to explain our theoretical study results of why the adhesion of real icing-rough solid interface shows strong, however, our theoretical values show weak. The key factor of mechanical interlocing from ice-solid interface is the main cause. Therefore, the adhesion work of ice-solid interface studied in our manuscrip only includes change in surface or interface tension, without considering the mechanical interlocking. [106] R. Johnson and R. H. Dettre, Contact angle, wettability, and adhesion. Advances in Chemistry Series 43(1964) 112. So to speak, this work is a pioneer study of contact angle, wettability, and adhesion. In our study, computation of contact angle, definition of wetting state, and change in surface free energy (or adhesion work), we completely adopted their idea apart from some method details. As a classically thermodynamic method, being widely acceptable, it can guarantee the effectiveness of our study. Note: The above mentioned serial numbers of references are in accordance with the ones in the original.
**Resource availability:**	No other resource being reserved.

## Method details

This study mainly focuses on the establishment of relationship between the anti–icing, icephobicity and microstructure, then to explain the physical nature of the anti–icing and icephobicity of the SHS, and to theoretically provide the reference for surface engineering. Our research mainly includes five aspects:

### How to model for the SHS

**Fig. 1:**
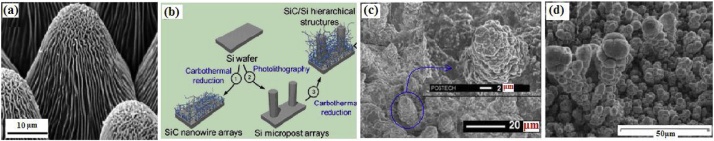
(a) The flower leaf of Rosa montana (adaxial side) with a rippled folded cuticle in the central field of the cells and parallel folding [1,2]; (b) A hierarchical SiC/Si nanostructure surface with underwater stability in superhydrophobicity, exhibiting obvious two-step micro/nano–structure [3]; (c) Photoresist replica of lotus leaf by UV–NIL [4]; (d) Diffusion–limited growth on surfaces with electrochemically deposited copper at 200 Ma cm^−2^ [5].

**Fig. 2:**
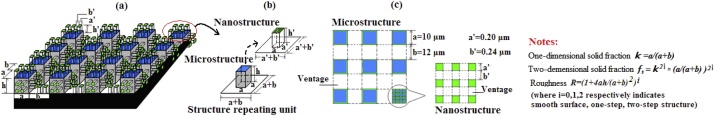
(a) An enlarged view of a typical pillar surface with two–step microstructure; (b) Structure repeating unit, limit the first step repeating unit in microscale, the second step repeating unit in nanoscale, the same step microstructural parameters (a,b,h) meet the ratios $\begin{array}{cc} \beta_{b}=1.2, & \beta_{h}=1.5 \end{array}$ if given width (a) of the first step pillar, the different step structural parameters (from a,b,h to a',b',h') meet the ratios $\beta =\frac{a}{a^{'}}=\frac{b}{{\text{b}}^{'}}=\frac{h}{h^{'}}=50$; (c) Vertical view of surface together definition of the related parameters (the concrete figure only for example).

We designed a 3–D model to simulate the system including a liquid water droplet→a frozen water droplet and the solid surface. Modeling background is based on the microstructure of natural and artificial SHS (Fig. 1) [[1], [2], [3], [4], [5], [6], [7], [8]], especially, a lotus surface with two–step micro/nanostructure, micro–hoodoos of the first step with mean diameter/spacing/height (a/b/h of 8–10/10–12/12–15μm) in micro–scale, and cylindrical wax tubules (with 150–200nmmean diameter as width (a') of the second step pillar) in nano–scale. Inspired by it, we define constant ratios $\beta_{b}=\frac{b}{a}=1.2,\;\;\;\;\beta_{h}=\frac{h}{a}=1.5$ as reference for design of the same step microstructure (spacing (b) and height (h)), if set width (a) of the first step micro–pillar in advance; and a ratio $\beta =\frac{a}{a^{'}}=\frac{b}{{\text{b}}^{'}}=\frac{h}{h^{'}}=\frac{10.00\mu m}{0.20\mu m}=50$, mean diameter ratio of a micro–hoodoo (in microscale) to a cylindrical wax tubule (in nanoscale) for design of the same step nanostructure. Considering the solid fraction (f_s_) and roughness (ϒ) as the average measurement of the surface microstructure, we constructed a two–step model (Fig. 2) to simulate the real surface with hierarchical structure by using a pillar–shaped microstructure, as the SHS for analysis of hydrophobicity of icephobicity, especially for the simplified calculation of the solid fraction (f_s_), the roughness (ϒ), and the other quantities, i.e., apparent contact angle (APCA) together contact angle hysteresis (CAH), and adhesion work (W_aw_, and W_ai_).

**Fig. 3:**
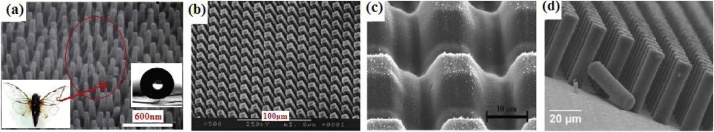
One–step microstructured surfaces with the superhydrophobicity. (a) FE–SEM micrograph of the cicada orni’s wing surface with regularly aligned nanoposts [9]; (b) Lithographic surface modification, photolithographic towers [12]; (c) Laser–modified SU_8_ surface [13]; (d) SU_8_ towers [14].

**Fig. 4:**
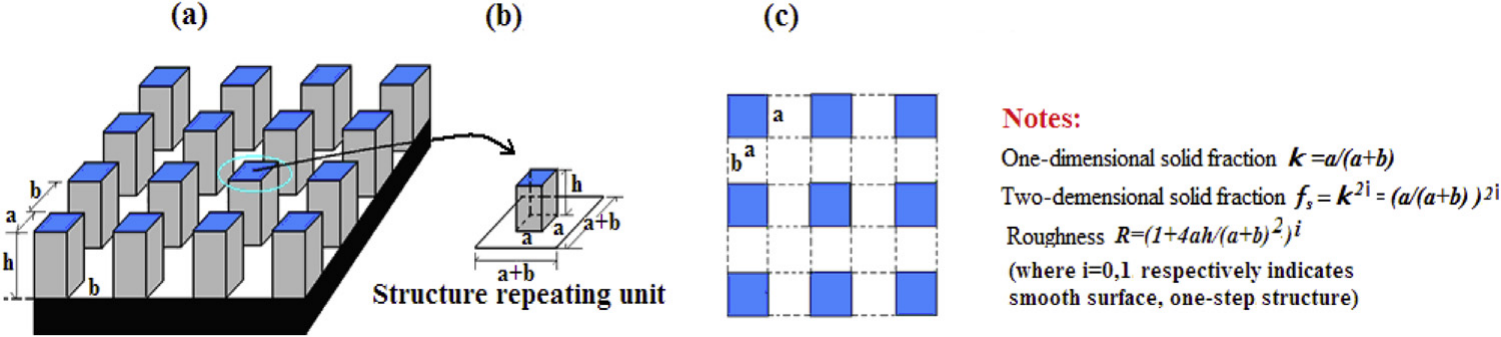
(a) An enlarged view of a typical pillar surface with one–step microstructure along with its (b) structure repeating unit; (c) Vertical view of surface together definition of the related parameters.

Although the lotus or lotus–simulating surface, as a typical composite structure, can efficiently increase the roughness, they are not only choice to create the superhydrophobicity. Some natural and artificial SHS justly have one–step structure with mean diameter/spacing/height in nano–scale [9,10] (Fig. 3). Referring to their micro–structure, in the same modeling way, a similar model for the SHS with one–step and pillar–shaped structure was also developed (Fig. 4), of which the scale is in micro– or nano–scale.

Our initial models are roughly based on the above design. Considering general micro–structure, we still gave their continuous change within limited scope (e.g., given b_1_=b = 2*10^−6^m, a_1_ and h_1_ continuously change within given scope) for the same step structure; but between the different steps, the simplification for the calculation of properties must abide by the same coefficient β=50based on self–similarity and fractal theory (Fig. 4) [11].

Some physical quantities studied in this paper, i.e., f_s_, ϒ, APCA(CA) together CAH, W_aw_, and W_ai_, all belong to the macro-characteristic of the wettability. For different microstructure and topography, if having the same solid fraction or roughness, the APCA together CAH, W_aw_, and W_ai_ possibly have the same values. Therefore, as a typical structure commonly used for simulation, such a pillar–type microstructure is still selected as a representative for our modeling, convenience and simplicity. Because the wettability is mainly determined by the surface roughness, solid fraction and the surface tension, study for the wetting property based on them, the corresponding result or conclusion of it may be regarded as generality for the other microstructure and topography.

### Computation for adhesion work (w_aw_) per unit area of a water droplet–SHS interface

**Fig. 5:**
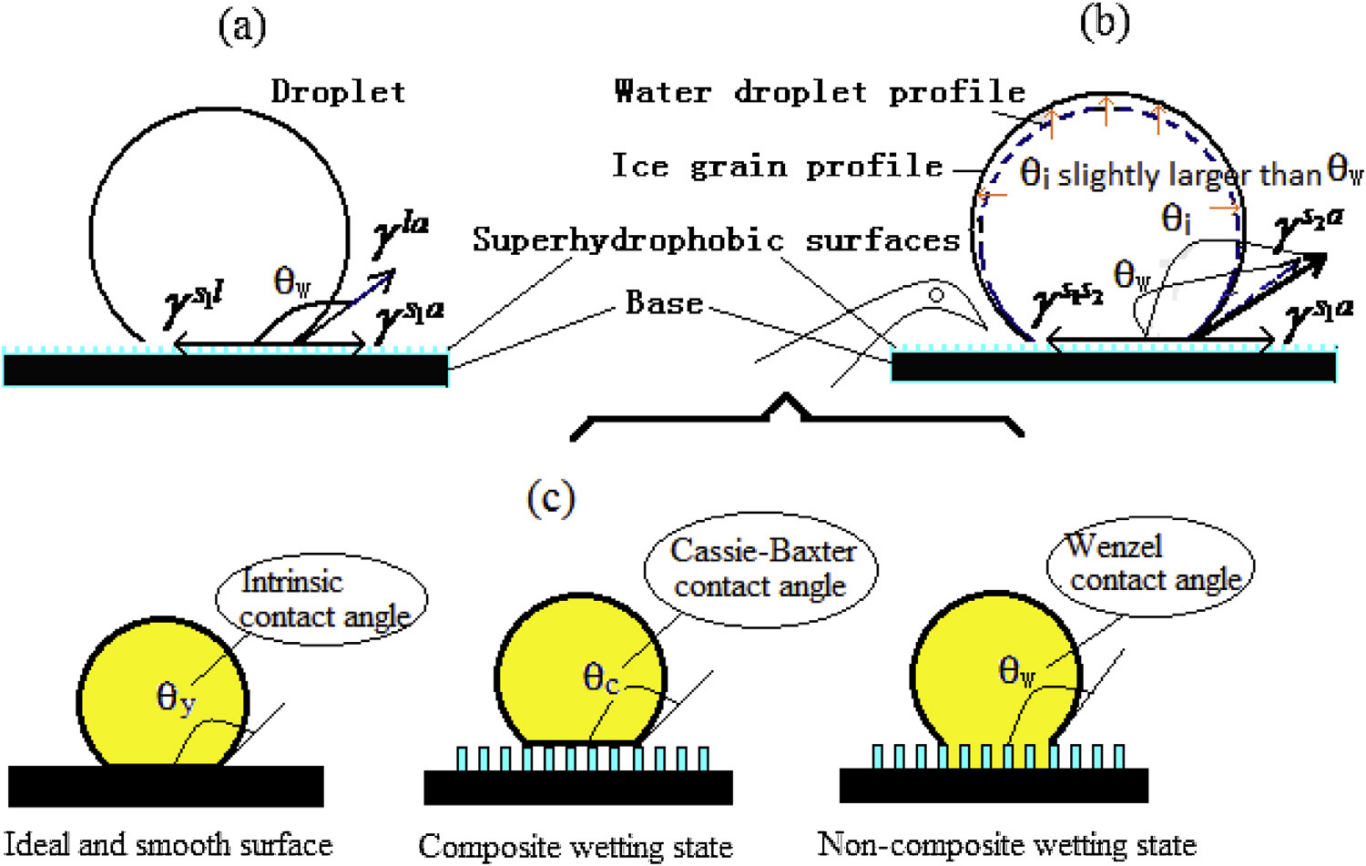
Stress state of a water droplet together with its frozen on the SHS. (a) Equilibrium state of a water droplet; (b) Change of the equilibrium state when a water droplet freezes, its volume dilate due to the change of molecular structure; (c) Composite and non–composite wetting states, ideal and smooth surface for reference.

Corresponding theoretical analysis for w_aw_ is the base of this study. Its main idea is based on the change in surface free energy (including solid–liquid interface energy), when a water droplet, together their frozen, contacts with the SHS (Fig. 5).

As can be seen from the Fig. 5(a), the adhesion work (w_aw_) per unit contact area of the water droplet–SHS interface is from the change in surface or interface free energy after contact of a water droplet with solid surface.

(1) $$w_{aw}=\gamma^{s_{1}a}+\gamma^{la}-\gamma^{s_{1}l}$$

Considering Young’s equation being only for the ideal and smooth surface, we have to modify and extend it to the real and rough surface, where the intrinsic CA (θ_y_) in it is substituted by the APCA (θ_w_) for the real surface, and the corresponding ideal surface tension ($\gamma^{sl}$,$\gamma^{sa}$)by the real surface tension($\gamma_{r}^{s_{1}l}$,$\gamma_{r}^{s_{1}a}$).

(2) $$\cos\theta_{w}=\frac{\gamma_{r}^{s_{1}l}-\gamma_{r}^{s_{1}a}}{\gamma^{la}}$$

Thus the Eq. (1) may be simplified as follows,

(3) $$w_{aw}=\gamma^{la}\left(1+\cos\theta_{w}\right)$$

By doing so, the adhesion work (w_aw_) of the water droplet–SHS interface can be obtained. In view of different wettability, composite and non–composite wetting states (CWS and NCWS) (Fig.5(c)), their APCA (θ_w_) are respectively determined by the Cassie–Baxter and Wenzel equations. Furthermore, the APCA (θ_w_) directly associated with these two equations are respectively related with the solid fraction (f_s_) and the roughness factor (ϒ). Thereby the relationship between the adhesion work (w_aw_) of the water droplet–SHS interface and surface micro–structure was established respectively by the solid fraction (f_s_) and the roughness (ϒ).

When a water droplet contacts with the solid surface, the total adhesion work (W_aw_) is equal to the product of the w_aw_ and the contact area between a water droplet and solid surface.

### Computation for adhesion work (w_ai_) per unit area of the frozen water droplet–SHS interface

This task is a difficult problem of the study, also a challenge to understanding of the adhesion work of the frozen water droplet–SHS interface. With the environmental temperature down to zero ℃, a water droplet starts to freeze. We think that, from a water droplet to its frozen, the computation of the adhesion work for a frozen water droplet should be operated in two stages, namely, contact of a water droplet with the SHS firstly, then the icing. The first stage corresponds to the adhesion work of a water droplet (W_aw_), the second stage to the reduced adhesion work (denoted as w_a2_) after icing, which is actually the change of the adhesion work. Therefore, the adhesion work from a frozen water droplet–the SHS interface may be presented in the following,

(4) $$w_{ai}=w_{aw}+w_{a2}=\gamma^{s_{1}a}+\gamma^{la}-\gamma^{s_{1}s_{2}}$$

(5) $$w_{a2}=\gamma_{r}^{s_{1}l}-\gamma^{s_{1}s_{2}}$$

Still referring to Young’s equation describing static equilibrium of a water droplet, we can obtain an equilibrium equation for the frozen water droplet (Fig. 5(b)),

(6) $$\gamma^{s_{1}s_{2}}=\gamma^{s_{1}a}-\gamma^{s_{2}a}\cos\theta_{i}$$

where θ_i_ represents CA for the frozen water droplet. Along with a water droplet frozen, it will dilate, resulting in the APCA for a frozen water droplet (θ_i_) lightly larger than one for a water droplet (θ_w_) (Fig. 5(b)). But its expanding rate is little due to density of mass being lightly decreased. Furthermore, during icing, the three phase contact line is not moved yet because of being pinned and increased CA not being up to advancing CA; thus the contact area does not change (in the next, the total contact area between a frozen water droplet and the SHS is equal to that between a water droplet and the SHS), and the APCA changes slightly. While computing, we may consider it proximately unchanged from liquid to solid state, namely θ_i_ may be substituted by θ_w_ only bringing little error to cosθ_i_. Then substituting Eq. (6) into (4), we may progressively obtain the following equation,

(7) $$w_{ai}=\gamma^{la}+\gamma^{s_{2}a}\cos\theta_{w}$$

At zero centigrade degree, the surface tension of a water droplet together its frozen is 75.64*10^−3^  J/m^2^ ($\gamma^{la}$), 82.00*10^−3^  J/m^2^ ($\gamma^{s_{2}a}$) respectively, thus Eq. (7) may be progressively simplified,

(8) $$w_{ai}=\gamma^{la}\left(1+\frac{\gamma^{s_{2}a}}{\gamma^{la}}\cos\theta_{w}\right)=75.64*\left(1+1.085*\cos\theta_{w}\right)$$

Note that calculation of w_ai_ indicates the theoretical values slightly higher than its real value due to substitution of θ_i_ by θ_w_ (θ_i_>θ_w_>150°). The less error does not affect reaching a conclusion. The above idea may be progressively explained in the following,

Firstly, as already pointed, we think, that adhesion work after icing should include two sections: one part is from the contact of a water droplet with solid surface (w_aw_), the other from the frozen droplet (w_a2_), because a water droplet being frozen actually involves two process of a water droplet contact with solid surface firstly and then the icing. Therefore, the w_a2_ from Eq. (4) is used for the second stage, of which a water droplet is frozen, namely transition from liquid to solid state. In view of this consideration, we still think that, the adhesion work, after icing, of unit area must be the sum of the above two part (as Eq. (4) illustrates) despite of w_a2_ being negative.

**Fig. 6:**
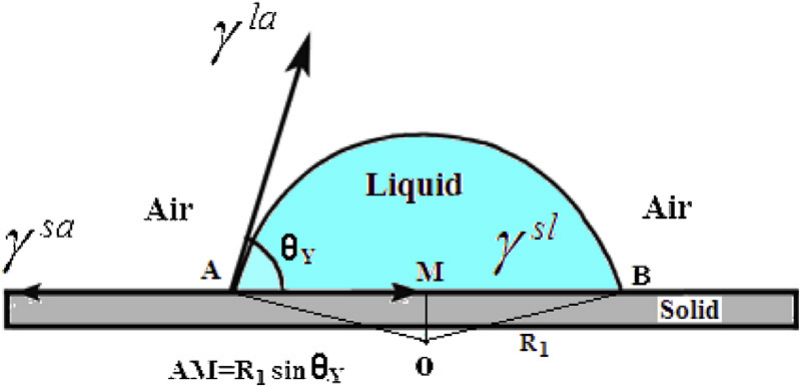
A droplet on the ideal and smooth surface with homogenous, flat and rigid structure, where $\theta_{Y}$ is the intrinsic CA only used for Young’s model, $\gamma^{sa}$,$\gamma^{sl}$,$\gamma^{la}$ are the surface tension of solid–air, solid–liquid, and liquid–air interfaces, respectively. The equation connects the interface tension with the intrinsic CA, suggesting the key role of the interface tension (as property parameter) to the intrinsic CA. if such a surface is not ideal and smooth (i.e., the superhydrophobic surface, being rough), the corresponding parameters must be substituted by real ones, i.e., $\theta_{Y}$ by $\theta_{A}$ (APCA), $\gamma^{sa}$, $\gamma^{sl}$ by $\gamma_{r}^{sa}$,$\gamma_{r}^{sl}$(real solid–air and solid–liquid interfacial tension) respectively.

Secondly, the transition from Eqs. (6), (7), (8), which is aimed to relate the adhesion work of a water droplet to the structured substrate, is based on the application of Young equation, however, of which the CA therein is the apparent contact angle (Cassie CA or Wenzel CA) for the real surface instead of Young contact angle (or the intrinsic CA). It is emphasized that, here, we only refer to the method of Young’s equation to describe the equilibrium state of a water droplet on the solid surface (as Fig. 6 indicates). Because of the SHS being rough, when a water droplet is statically deposited on such surface, the associated CA in the equilibrium equation should be the APCA instead of intrinsic CA. The concrete operation is as follows,

(1) when the solid surface is ideal, smooth, rigid and homogenous, Young’s equation may be used for description of equilibrium state in the horizontal direction, indicating the relationship between surface tension and intrinsic CA.

(9) $$\gamma^{sa}=\gamma^{sl}+\gamma^{la}\cos\theta_{Y}$$

(2) For the real solid surface, i.e., the SHS, being commonly rough, we may properly modify Young’s equation, similarly to describe the equilibrium state, e.g., intrinsic CA (θ_y_) is substituted by the APCA (θ_w_), $\gamma^{sa},\gamma^{sl}$ by real solid-air, solid-liquid interface tension ($\gamma_{r}^{sa},\gamma_{r}^{sl}$).

(10) $$\gamma_{r}^{sa}=\gamma_{r}^{sl}+\gamma^{la}\cos\theta_{A}$$

The above contents refer to our previous study [[15], [16], [17], [18]].

Thirdly, we originally take into account that the CAs respectively for the frozen water droplet and the water droplet are different. Fig. 5(b) reveals the expanded state after the water droplet freezes. Only the difference between them is little, we still use the same θ_w_ to indicate.

### Computation for reduced adhesion work (w_a2_) per unit area of the frozen water droplet–SHS interface

By Eq. (5), it is difficulty to directly conduct the computing of the reduced adhesion work (w_a2_) because of unknown $\gamma_{}^{s_{1}l}$ and $\gamma^{s_{1}s_{2}}$. We only obtained it indirectly by computing$w_{aw}andw_{ai}$, then using Eq. (4) to compute $w_{a2}=w_{ai}-w_{aw}$. By visualization of w_a2_, the decrease of the adhesion work after icing was further confirmed.

### Hydrothermal experiment together others, and validation between model and experimental results

**Fig. 7:**
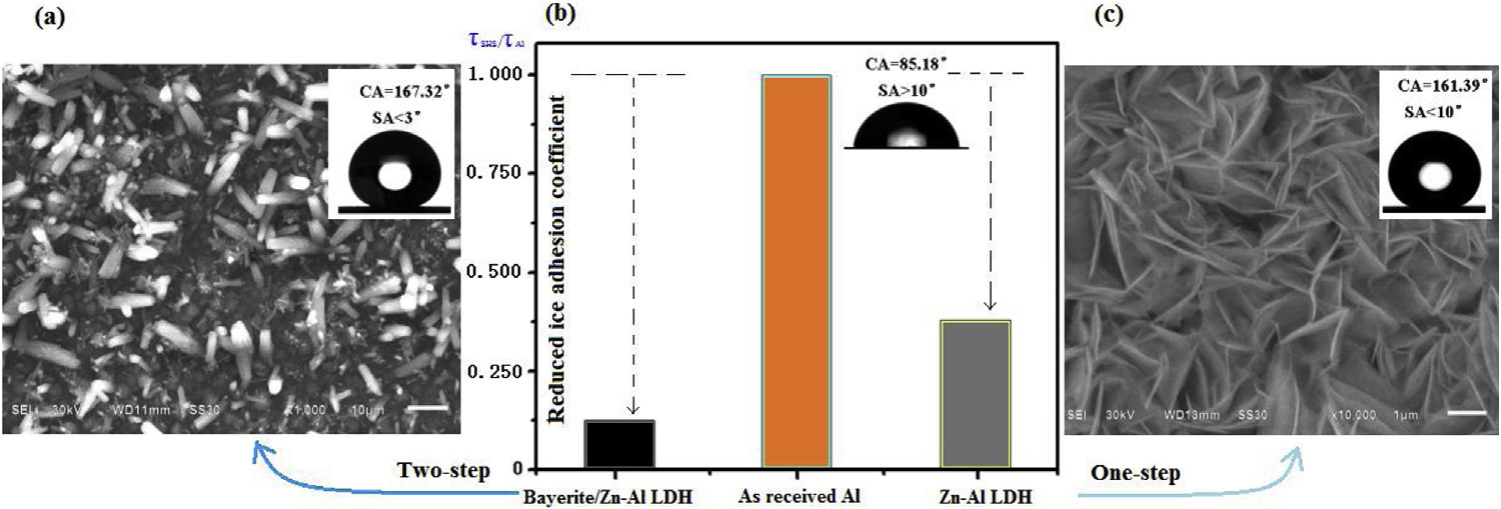
Comparison of reduced ice adhesion coefficient, along with special micro–topography, as well as hydrophobic property of the surface, (a) SEM of Bayerite/Zn–Al LDH surfaces with hierarchical structure (two–step), (b) Comparison of reduced ice adhesion coefficient, (c) SEM of Zn–Al surface with nanoscale walls (one–step).

As a proof experiment, by using hydrothermal method, X . K. Zhang respectively obtained the Zn–Al LDH (Layered Double Hydroxide) superhydrophobic films with layer–shaped nanowalls and interconnected hollow structure (static water APCA up to 161.39°, SA< 10°) [19], as well as the Bayerite/Zn–Al LDH superhydrophobic films with micro/nano composite structure (static water CA up to 167.32°, SA < 3°), respectively in molarity rate of NH_3_H_2_O/Zn^2+^ of 0.75:1 and 1.25:1 (Fig. 7). Followed by it, the icing experiments were conducted at –10 °C respectively based on the Zn–Al, Bayerite/Zn–Al LDH surface, together as–received Al surface for reference. Their delay time of icing are 249 s, 380 s respectively relative to the as–received Al surface, the reduced adhesion coefficient (τ_SHS_/τ_Al_, rate of shear forces of the icing between the SHS and the as–received Al surface) declined to 0.33 and 0.125, respectively, representing a significant role of the SHS with special topography in delaying the freezing time [20,21]. Meanwhile, we also referred to parts of experiment result of Yang’s and Miao’s. For example, Yang fabricated the SHS for anti–icing by chemical deposition, etching, modified polymer; Miao also obtained the similar SHS by hydrothermal experiment [22,23]. Based on these experiment we may acquire the supporter of our theoretical analysis, namely, parts of superhydrophobic surface with hierarchical structure and specific topography surely have such property to anti–icing [24].

For validation and help to understand of modeling, we listed our research team member’s ( X . K. Zhang, J.yang, and F.H. Miao) experimental results for comparison with model’s prediction aiming at composite wetting state.

Summarizing the above comparison, generally speaking, for composite wetting state, the model prediction conforms to the experimental results, by which our modeling line is reasonable, thus adaptable, being capable of predictability.

**Fig. 8:**
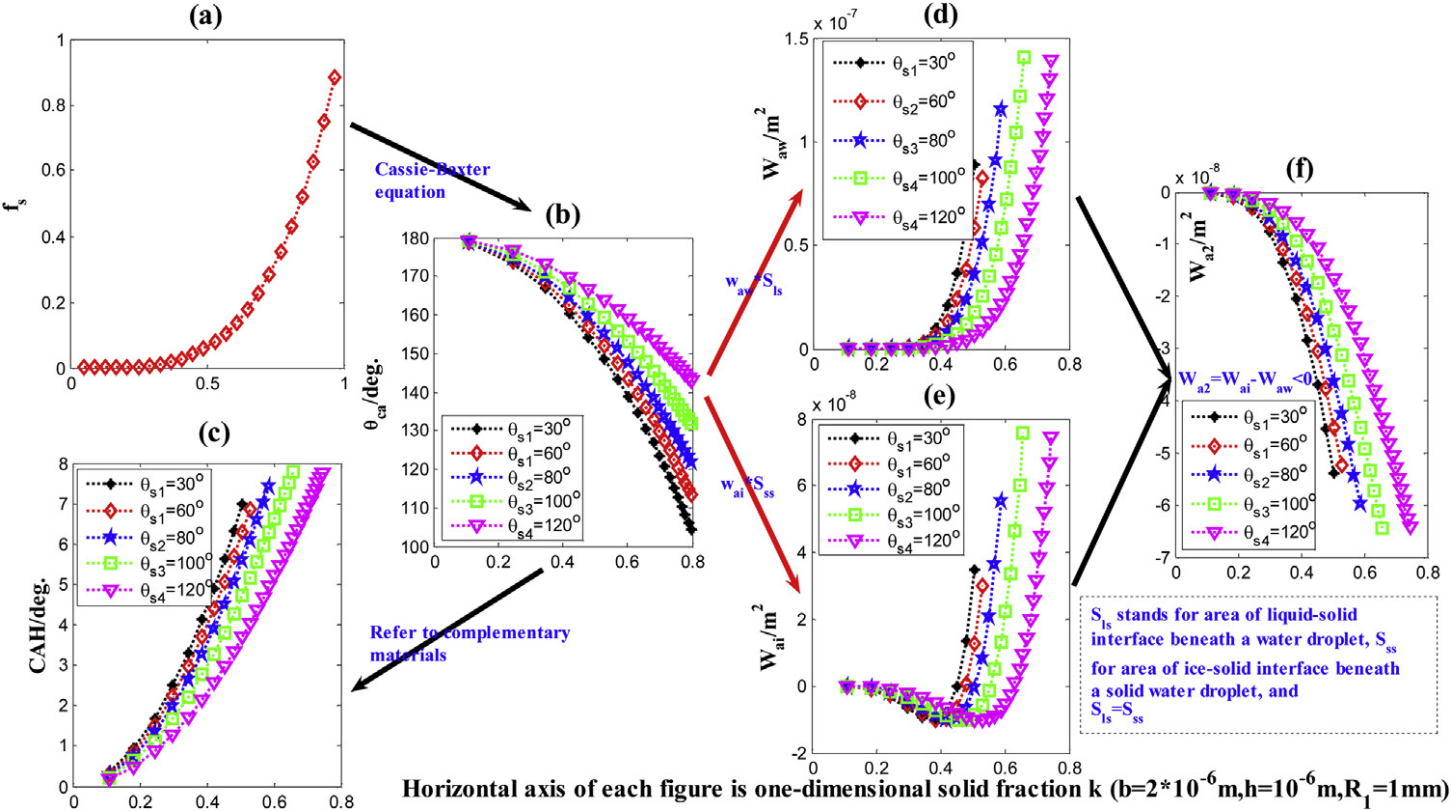
Variations of the properties of the two–step surface with one–dimensional solid fraction (κ) in composite wetting state (CWS). (a) The solid fraction (f_s_); (b) Cassie’s contact angle (θ_cs_); (c) Contact angle hysteresis (CAH); (d) and (e) Normalized total adhesion work of a water droplet and its frozen respectively from the liquid-solid and solid-solid interfaces (W_aw_ and W_ai_);(f) Reduced adhesion work after icing.

For the non–composite wetting state, although we have no adaptable instrument to measure the roughness accurately, at least, model prediction can qualitatively explain series of experiment results on the SHS for anti–icing/icephobicity, of which the SHS, with hierarchical micro/nanostructure and low surface energy coatings(solid–liquid interface often appears composite wetting state), have fewer adhesion force (the reduced quantity is up to 90%)and long icing delay time compared with the smooth surface (solid–liquid interface appears non–composite wetting state) [[29], [30], [31]]. According to our computing (compare Fig. 8(e) for composite with 9(d') for non–composite), if from the adhesion work, for the same solid water droplet, the W_ai_ from composite wetting state is obviously fewer than that from non–composite wetting state, even having difference in order of magnitude (10^-−8^ vs. 10^-6^). In consideration of different contact area, if transform the ratio of the adhesion work into that of the adhesion force, the ratio of the adhesion force is closer to the above reality [[29], [30], [31]].

**Fig. 9:**
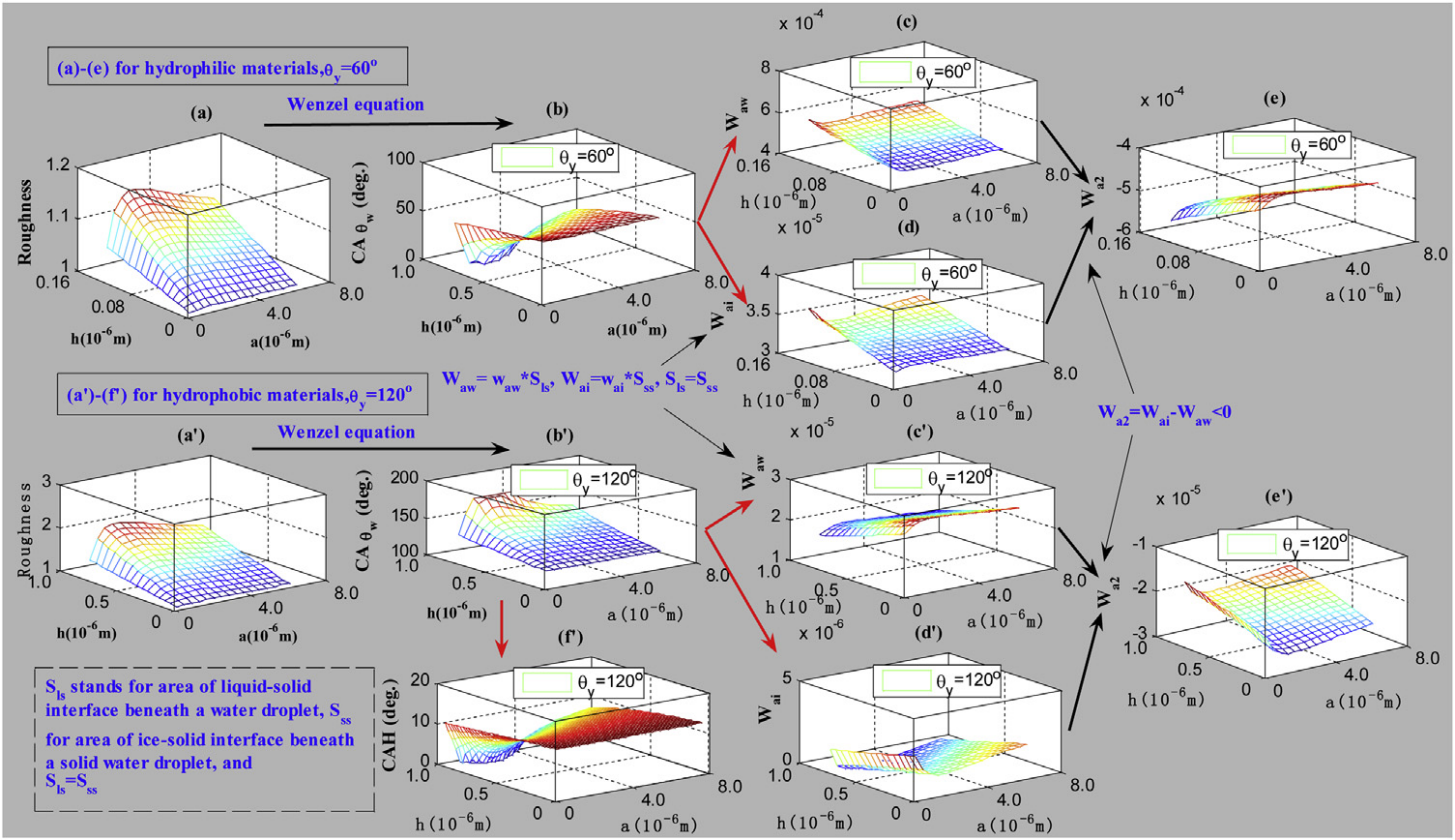
Variations of the properties of the two–step surface with the microstructure or roughness (width (a), height (h)) at constant spacing (b = 2*10-6 m) of a pillar in non–composite wetting state. (a) Roughness for reference of the hydrophilic materials (i.e., θy = 60°, as reference for (b), (c), (d), (e)); (b) Wenzel’s contact angles; (c) Normalized adhesion work of a water droplet; (d) Normalized adhesion work of a frozen water droplet; (e) Reduced normalized adhesion work after icing; (a′) Roughness for reference of the hydrophobic materials (i.e.,θ_y_ = 120°, as reference for (b′), (c′), (d′), (e′),(f′)); (b′) Wenzel’s contact angles; (c′) Normalized adhesion work of a water droplet; (d′) Normalized adhesion work of a frozen water droplet;(e′) Reduced normalized adhesion work after icing; (f′) Contact angle hysteresis.

Taken all together, the selected experiment results can prove the validation of model to some extent for special surface topography, in spite of not all the SHS having excellent anti–icing/icephobicity property due to abrasion, ageing, and weak mechanic property [[32], [33], [34]]. Moreover, although we can not quantitatively control the microstructure scale arbitrarily, the prepared surfaces have microstructure (i.e., Table 1. rows (2) and (4)) roughly within the bounds of modeling with the first micropillar having microscale (0.2 μm < a<8 μm, based on 0.1<κ<0.8, see Fig. 8, Fig. 9), and the second nanopillar having nanoscale (4 nm < a'<160 nm, based on $\beta =\frac{a}{a^{'}}=50$). Originally our modeling is based on the natural and artificial SHS, thus not resulting in a big gap.

**Table 1:** Comparison between model prediction and experimental results.

Experimental preparation	Hierarchical structure and micro/nano scale	Hydrophobicity (characterized by CA and SA)	Anti-icing or icephobicity(delay icing time or adhesion effect)	Corresponding theoretical prediction (CA,CAH or W_ai_) based on the same microscale and hierarchical structure	Review on the consistency between experiment and theoretical prediction
(1) Zn–Al LDH superhydrophobic films with layer–shaped nanowalls and interconnected hollow structure by hydro-thermal method [20,21]	One–step microstructure. 50 nm of the nanowalls average thick, and 5–20 μm of diameter of nanotubes	CA = 161.39°, SA<10°, Based on the intrinsic CA θ_s_ = 109° of the as–received Al surface coated with stearic acid [25], approximately find f_s_ = 4.82%.	The reduced adhesion coefficient (τ_SHS_/τ_Al_) is 0.33, say, the adhesion of the icing to the thin films is nearly one third of that to the as-received Al surface.	By f_s_ = 4.82%, find κ = 0.2195.Then based on Cassie–Baxter equation and Eq. (18) in the supporting information further find CA = 161.2°or so, CAH<5°, thus SA<5°(SA < CAH) necessarily.	For CA, its experimental value is close to the theoretical value. For SA, its experimental result is beyond the range of the theoretical prediction.
(2) Bayerite/Zn–Al LDHsuperhydrophobic films with micro/nano composite structure [20,21]	Two–step microstructure. 50 nm of the nanowalls average thick for the second step nanostructure, and 5–20 μm of diameter of microrods for the first step microstructure	CA = 167.32°, SA<3° (by X . K.Zhang’s measurement and analysis, θ_s_ = 109°, f_s_ = 3.62%).	The reduced adhesion coefficient (τ_SHS_/τ_Al_) is 0.125, say, the adhesion of the icing to the thin films is nearly one eighth of that to the as-received Al surface. Comparing (1) with (2) rows, we can find that (τ_SHS_)_one-step_/ (τ_SHS_)_two-step_ = 0.33/0.125 = 2.64	Compared with the left, by f_s_ = 3.62%, find κ = 0.1903. Then based on Cassie–Baxter equation and Eq. (18) in the supporting information further find CA = 175°or so, CAH<1°, thus SA<1° necessarily. Meanwhile, we also find that (W_ai_)_one-step_/ (W_ai_)_two-step_<18, subsequently infer (τ_SHS_)_one–step_/ (τ_SHS_)_two-step_ < 7.2 (here refer to κ = 0.6, θ_s_ = 100° due to κ = 0.1903 having (W_ai_)_two-step_<0)	For property parameters CA,SA, (τ_SHS_)_one-step_/(τ_SHS_)_two-step_, the experimental results is less than that of the theoretical prediction, but for SA being justly conversely.
(3) Preparation of superhydrophobic surfaces by chemical deposition based on aluminum alloy surface, then modified with stearic acid solution (8 mol/L) [22].	Approximately one–step microstructure. Grating texture, characteristic scale less than 5μm	CA< = 163°, SA=?(untested) Smooth stearic acid surface of intrinsic CA (θ_s_) is approximately 109° [25]. By SEM image, find f_s_ = 6.76% or so, thus κ = 0.26 or so.	Not selected for the icing experiment	Using κ = 0.26, θ_s_ = 109°, by our model and computing, find CA = 161° or so, CAH<5°, thus SA<5° necessarily. for one-step.	For CA, theoretical prediction from hydrophobic model largely accords with experiment results.
(4) Preparation of of aluminum alloy Superhydrophobic surface by one–step etching method,then modified with fluorine silicane [22]	Grid–shaped multi–layered structure,1–2 μm of grid width, 3–4 μm of grid depth.	CA = 167°, SA<5°.By SEM image, find f_s_ = 16% or so, thus κ = 0.4 or so. And note for fluorine silicane, θ_s_ = 101° [26].	At -10℃,600–700 s of delayed icing time. The quantity of icing on the specimen decreases with the increasing tilt angles. At -6–-9℃, 400 s of delayed icing time when the coatings used for high voltage cables.	Using κ = 0.4, θ_s_ = 101°, by our model and Cassie–Baxter equation, find CA = 155° or so, CAH<8°, thus SA<8° necessarily for one–step.	For CA and SA, model prediction and experiment results are close to each other. For delayed icing time, model has no way to quantitatively predict. But at least it may qualitatively predict the long icing time because of the large liquid–air contact compared with the smooth surface or low rough surface.
(5) Preparation of of polystyrene superhydrophobic surface [22]	Chip–shaped, multi–layered, and branched chain structure with multi–holes, characteristic scale of microscale	CA>150°, SA=?(untested), Via checking, smooth polystyrene surface of intrinsic CA is approximately 89°(namely θ_s_ = 89°) [27]. Then infer f_s_<13.17%, thus κ < 0.363.	Not selected for the icing experiment	By κ < 0.363, theoretically inferring CA>152°, CAH<8°,thus SA<8° for one–step.	For CA, theoretical estimation generally accords with experimental value.
(6) Preparation of of phenolic resin superhydrophobic surface [22]	Irregular block shape microstructure with holes, tens of microns of diameter, and 10 μm (or so) of thickness	CA close to160°, SA=?(untested).Like-wise, via checking, smooth polystyrene surface of intrinsic CA is approximately 95°(namely θ_s_ = 95°) [28]. Then infer f_s_≈6.61%, thus κ≈0.2571.	Not selected for the icing experiment	By κ < 0.2571, theoretically inferring CA≈160°, CAH<5°,thus SA<5° for one–step.	For CA, theoretical estimation generally accords with experimental value.
(7) Nickel hydroxide (Ni(OH)_2_) coatings based on the glass substrate by hydro-thermal method, then modified with an ethanol solution of stearic acid [23]	Two–step Stratified microstructure with multi–holes in nanoscale	CA up to 168.45°, SA<5°, and f_s_ = 3.0％, thus κ = 0.1732.	At -10℃,350–600 s of delay icing time based on different volume of water droplet. The quantity of icing on the specimen varies with the temperature and time as well as height of dripping.	Using κ = 0.1732, θ_s_ = 109°, by our model and Cassie-Baxter equation, find CA<178° or so, CAH<1°, thus SA<1° necessarily. for two-step.	For CA and SA, there exists some difference between model prediction and experiment results.But experiment results are still close to theoretical prediction. Similarly, for delayed icing time, in spite of our model having no way to quantitatively predict, it still may qualitatively predict the long icing time because of the same cause.

## Results and discussion

Based on the theoretical analysis (Eqs. (3),(8) for the adhesion work), by computing we obtained the corresponding macro-properties. As an example, we mainly listed the results of the computation for the two–step micro–structure, respectively aiming at the composite and non–composite wetting states.

For the CWS, the solid fraction (two–dimension, f_s_) refers to the notes of Fig. 2; the computation of the APCA is based on Cassie–Baxter Equation; then according to the obtained CA, the CAH’s computation refers to our previous study or complementary materials [[15], [16], [17], [18]]. More importantly, the computation for the key property of the total adhesion work (W_aw_ and W_ai_) directly uses Eqs. (3) and (8), but both the APCA and the contact area of liquid–solid interface must be known, i.e., the W_aw_ is equal to the product of the w_aw_ and contact area of liquid–solid interface, likewise, the W_ai_ to the product of the w_ai_ and contact area of solid–solid interface (where the two area is equal). The above properties all are as the functions of the one–dimensional solid fraction (κ), by which we may make a detail analysis based on their visualization (Fig. 8), i.e., how those properties are determined by the micro–structure, relationships between the icephobicity/superhydrophobicity and materials (denoted as different intrinsic CA, θ_y_ or θ_s_) or micro–structure. Our previous study for Physicochemical mechanism of the SHS was mainly based on such line which will continuously guide our future research [[15], [16], [17], [18]].

Meanwhile, for the NCWS (Fig. 9), the roughness (two–dimension, ϒ) also refers to the notes of Fig. 2; the computation of the APCA is based on Wenzel Equation; then according to the obtained APCA, the CAH’s computation still refers to our previous study or complementary materials [[15], [16], [17], [18]]. Furthermore, the computation for the key property of the total adhesion work (W_aw_ and W_ai_) directly makes use of Eqs. (3) and (8), but both the APCA and the contact area of liquid–solid interface also must be known as the above explains. The same question for the NCWS may be discussed again.

Moreover, for one–step structure, the same work has been conducted. Therefore, we may further analyse such question as below, i.e., how those properties are determined by the dimension of micro–structure, if there exist the intrinsic difference between the one–step and the two–step structure. Then what relationship exists between the CWS and the NCWS on superhydrophobicity, as well as on icephobicity. To the end, we can further solve those questions, for these factors, i.e., the dimension, materials, CWS and NCWS, how they affect the icephobicity or anti–icing of the SHS.

**Fig. 10:**
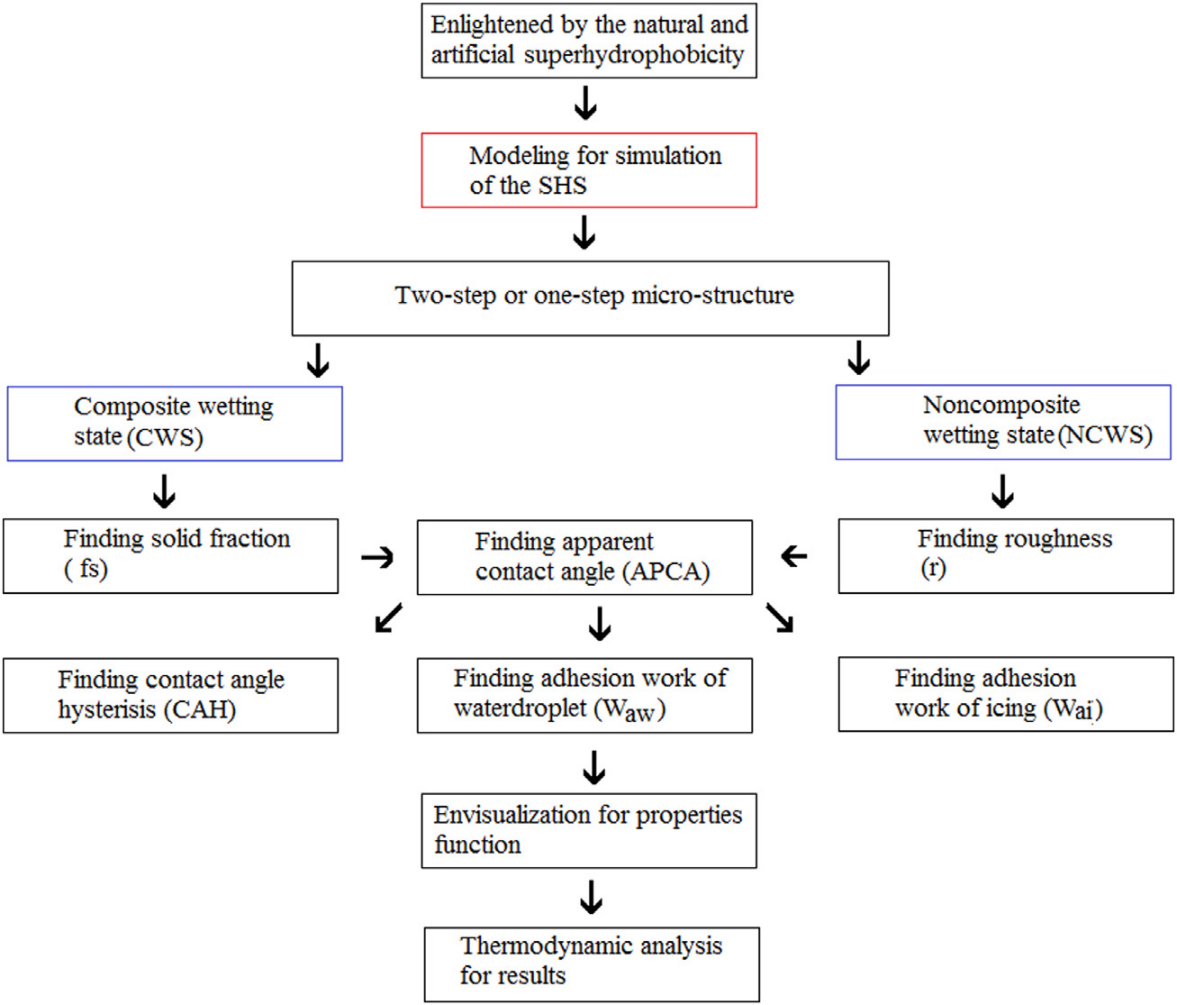
Flow chart of study for comparison between icephobicity and superhydrophobicity.

Summarizing the whole study process, we may outline the route diagram as follows (Fig. 10), which presents our study the systematicness and logic.
